# Effect of Acute Hyperglycemia on Left Ventricular Contractile Function in Diabetic Patients with and without Heart Failure: Two Randomized Cross-Over Studies

**DOI:** 10.1371/journal.pone.0053247

**Published:** 2013-01-08

**Authors:** Roni Nielsen, Helene Nørrelund, Ulla Kampmann, Hans Erik Bøtker, Niels Møller, Henrik Wiggers

**Affiliations:** 1 Department of Cardiology, Aarhus University Hospital, Aarhus, Denmark; 2 Department of Medicine, Viborg Hospital, Viborg, Denmark; 3 Department of Endocrinology and Metabolism, Aarhus University Hospital, Aarhus, Denmark; Universita Magna-Graecia di Catanzaro, Italy

## Abstract

**Background:**

It is unknown whether changes in circulating glucose levels due to short-term insulin discontinuation affect left ventricular contractile function in type 2 diabetic patients with (T2D-HF) and without (T2D-nonHF) heart failure.

**Materials and Methods:**

In two randomized cross-over-designed trials, 18 insulin-treated type 2 diabetic patients with (Ejection Fraction (EF) 36±6%, n = 10) (trial 2) and without systolic heart failure (EF 60±3%, n = 8) (trial 1) were subjected to hyper- and normoglycemia for 9–12 hours on two different occasions. Advanced echocardiography, bicycle exercise tests and 6-minute hall walk distance were applied.

**Results:**

Plasma glucose levels differed between study arms (6.5±0.8 mM vs 14.1±2.6 mM (T2D-HF), 5.8±0.4 mM vs 9.9±2.1 mM (T2D-nonHF), p<0.001). Hyperglycemia was associated with an increase in several parameters: maximal global systolic tissue velocity (Vmax) (p<0.001), maximal mitral annulus velocity (S'max) (p<0.001), strain rate (p = 0.02) and strain (p = 0.05). Indices of increased myocardial systolic contractile function were significant in both T2D-HF (Vmax: 14%, p = 0.02; S'max: 10%, p = 0.04), T2D-nonHF (Vmax: 12%, p<0.01; S'max: 9%, p<0.001) and in post exercise S'max (7%, p = 0.049) during hyperglycemia as opposed to normoglycemia. LVEF did not differ between normo- and hyperglycemia (p = 0.17), and neither did peak exercise capacity nor catecholamine levels. Type 2 diabetic heart failure patients' 6-minute hall walk distance improved by 7% (p = 0.02) during hyperglycemia as compared with normoglycemia.

**Conclusions:**

Short-term hyperglycemia by insulin discontinuation is associated with an increase in myocardial systolic contractile function in type 2 diabetic patients with and without heart failure and with a slightly prolonged walking distance in type 2 diabetic heart failure patients. (Clinicaltrials.gov identifier NCT00653510)

## Introduction

Epidemiological observations [1] suggest a causal relation between type 2 diabetes (T2D) and the development and progression of heart failure (HF). Furthermore, in both HF and T2D, whole body metabolism is characterized by increased levels of circulating glucose, free fatty acids (FFA), and insulin [2], [3]; and the combination of HF and T2D induces complex metabolic changes in the myocardium [4]. It is a matter of debate whether these abnormalities [3], [5], [6] are causally involved in the progression of HF or merely epiphenomenal [7], [8].

Current literature is inconsistent as to the effect on HF of therapy aimed at optimising glycemic control in T2D patients [9], [10]. Randomized clinical trials evaluating the optimal glycemic level in T2D HF patients are lacking. Data from the UKPDS [10] trial showed that the risk of developing HF rose with increasing HbA1c in T2D patients. In contrast, the ACCORD trial [9] reported a significant increase in fluid retention and a non-significant increase in incident HF among T2D patients on strict glycemic control. Previous studies have investigated the effect of myocardial glucose (MGU) and FFA uptake modulation on left ventricular function by euglycemic hyperinsulinemic clamping or by reducing circulating FFA. However, these results have been conflicting [11]–[14], and knowledge about the cardiovascular effects of different glucose levels in HF and T2D patients remains scarce.

We hypothesized that short-term hyperglycemia by lowered insulin treatment as opposed to normoglycemia would have detrimental effects on left ventricular contractile function in T2D patients. Thus, in the present open-labeled, randomized cross-over-designed trials we aimed to investigate the cardiovascular effects of short-term hyperglycemia induced by insulin discontinuation as opposed to normoglycemia in T2D patients, and whether patients with HF responded differently than patients without HF. We studied left ventricular systolic and diastolic function by 2D- and tissue-Doppler echocardiography, hemodynamics, exercise capacity, and hall walk test. We found that insulin discontinuation is associated with an increase in myocardial systolic contractile function in T2D patients with and without HF and with prolonged walking distance in T2D-HF patients.

## Methods

### Ethics

The present investigation was conducted as two separate trials for safety reasons. We sought to determine whether short term hyperglycemia could cause clinical adverse effects (shortness of breath, dizziness etc.) before subjecting HF patients to these conditions. Moreover, we chose to investigate the HF patients in our Department of Cardiology, Aarhus University Hospital, Aarhus, Denmark with staff specialized in cardiology if any severe adverse effects should occur. The T2D patients without HF (T2D-nonHF) were investigated at the Department of Endocrinology and Metabolism. Thus, we enrolled T2D-nonHF patients between 2008 and 2010 in one trial, whereas T2D-HF patients were enrolled during 2010 in a second trial. The interventions and examinations described below were performed similarly in patients regardless of LVEF unless stated otherwise. However, additional investigations of whole-body and muscular metabolism were performed in the T2D-nonHF patients and have been published elsewhere [15]. Due to these circumstances the present studies are registered under two different identifiers at http://www.clinicaltrial.gov (http://www.clinicaltrials.gov/ct2/show/NCT00653510 (referring to the T2D-nonHF patients) and http://www.clinicaltrials.gov/ct2/show/NCT01071772 (referring to the entire study population i.e. T2D-HF patients and the T2D-nonHF patients enrolled in NCT00653510)). They were approved by the Central Denmark Region Committees on Health Research Ethics, informed written consent was obtained from each patient, and the trials were conducted according to the protocols. These protocols and supporting CONSORT checklist are available as supporting information; see Checklist S1 and Protocol S1.

### Patients

We included 20 insulin-treated T2D patients. Ten had HF (T2D-HF) due to chronic ischemic heart disease, were stable on optimal HF medication, in New York Heart Association (NYHA) class 2–3, and had LVEF ≤45% as measured by echocardiography. Another ten of the patients had no prior cardiovascular events (admissions, interventions or other treatments etc. for cardiac disease) and their left ventricular ejection fraction (LVEF) was >50% as measured by echocardiography (T2D-nonHF). We excluded patients with high age (>80 years), cardiac valve disease, physical or psychological disability, creatinine >220mikr.M, alanine aminotransferase >3 fold the upper normal limit, or myocardial infarction within the past 3 months.

### Design

Patients were investigated after 9 (T2D-HF) to 12 (T2D-nonHF) hours of hyperglycemia and normoglycemia on two different occasions 2–6 weeks apart in a randomized, open–labeled (i.e. the randomization was not concealed), 2×2 cross-over-design (i.e. all patients were subjected to both hyper- and normoglycemia in each of the two trials). Patients were enrolled, randomized and allocated 1∶1 by the investigators to either hyperglycemia followed by normoglycemia or, opposite, by drawing from sealed envelopes before attending the two visits. The investigators were blinded to the randomization of the patients during data analysis.

**Figure 1:**
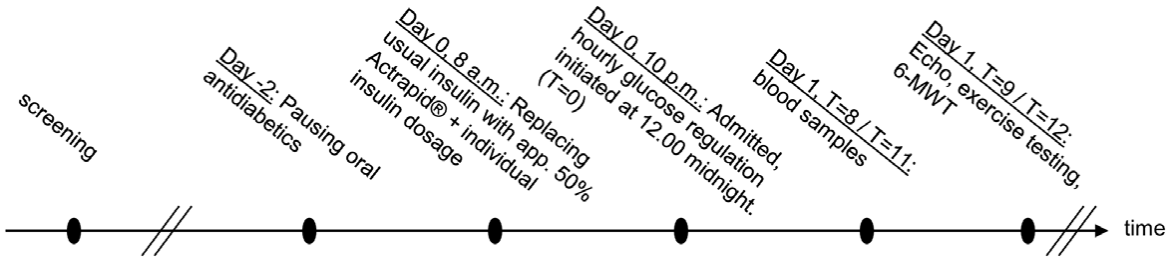
Timeline. All enrolled patients were investigated on two different occasions with similar timelines. They paused oral antidiabetics (day −2), were instructed to take individual insulin dosage (day 0) and admitted at 10 p.m. Hourly glucose measurements and adjustments were initiated at 12.00 midnight (T = 0), blood samples were drawn at T = 8 (T2D-HF) or T = 11 (T2D-nonHF) and echocardiography (echo) followed by exercise test, post exercise echo and 6-minutes hall walk test (6-MWT) at T = 9 (T2D-HF) or T = 12 (T2D-nonHF).

All patients were instructed to pause oral antidiabetic medication 2 days prior to admittance. They reduced their usual insulin dosage by approximately 50% on the day of admittance and replaced it with short-term acting insulin (Actrapid, Novo Nordisk) (figure 1). Patients were informed to take additional Actrapid units on an individual basis depending on their blood glucose levels and the planned glucose level during the investigations. Patients were admitted at 10 p.m. and venous cannulas were inserted into each of their upper extremities for infusion and blood sampling. After 9 to 12 hours of normo- or hyperglycemia, the patients underwent echocardiography at rest, 6-minute hall walk test, exercise testing, and post-exercise echocardiography.

### Normoglycemic situation

At midnight, the patients started fasting and Actrapid infusion was initiated to lower and to keep their plasma glucose at 5–7 mM. Blood was drawn from the opposite arm for plasma glucose analysis. Simultaneous glucose infusion was avoided. Measurements were performed every hour as a minimum.

### Hyperglycemic situation

Actrapid infusion was initiated if glucose levels increased to more than 20 mM. Glucose infusion was not used to raise the plasma glucose levels.

### Blood samples

Plasma glucose measurements were performed using HemoCue Glucose 201 (Ängelholm, Sweden) which measures whole blood glucose and calculates plasma glucose (T2D-HF) or Beckman (Coulter, Palo Alto, CA) (T2D-nonHF). The Beckman and HemoCue have been shown to produce similar results [16]. All blood samples were drawn prior to the investigations (figure 1), immediately cooled, spun, and stored at −80° Celsius until analysis. We analyzed the levels of FFA, insulin, C-peptide, glucagon, cortisol, growth hormone, ghrelin, and adiponectin as previously described [2]. Estimated glomerular filtration rate (eGFR) was calculated using the MDRD formula [17]. Adrenaline and noradrenaline were analyzed by a valid in-house time-resolved high-performance liquid chromatography method. Beta-hydroxybutyrate was analyzed by a commercial amperiometric method (Randox Analysis Kit RB1007, Abbott) as previously described [2] and N-Terminal pro-Brain Natriuretic Peptide (NT-proBNP) using a commercial electrochemiluminescent assay (Roche Diagnostics, Denmark) as described by the manufactures.

### Echocardiography

Echocardiographic measurements were performed at 9 a.m. (T2D-HF) or 12 a.m. (T2D-nonHF) by one observer on either a “Vivid Seven” (T2D-HF) or a “Vivid Five” (T2D-nonHF) ultrasound scanner (GE Medical System, Horten, Norway) with a 2.5-MHz transducer. EchoPAC 9 analysis software (GE-Vingmed Ultrasound, Horten, Norway) was used for analysis. We applied the Simpson's biplane method and measured the endocardial border during end diastole (EDV) and systole (ESV) and calculated LVEF = (EDV-ESV)/EDV. Using tissue-Doppler imaging, we measured global myocardial peak systolic velocity (Vmax), peak systolic strain rate (myocardial deformation rate), and strain (myocardial longitudinal deformation in percentage) in the mid-ventricular and basal segments, and longitudinal mitral annular peak velocity (S'max) during the ejection period as previously described by measuring the velocity of the mitral ring displacement [14], [18]. Measurements were performed at rest and after maximal exercise. We assessed left ventricular diastolic function from mitral inflow and tissue-Doppler: E-wave (early diastolic filling), A-wave (late diastolic filling), E/A-ratio, and mitral plane e' velocity (tissue velocity of the mitral ring during early diastole). These parameters were estimated as averages of either three (sinus rhythm) or five (atrial fibrillation) consecutive heart beats. The functional longitudinal reserve index (i.e. ability to increase diastolic and systolic function from rest to exercise) was calculated as previously described [19].

### Exercise testing

Patients performed a staged bicycle exercise test where the stages either lasted 1 minute and increments were in the order of 10 watts/min (T2D-HF) or lasted 2 minutes and increments were in the order of 25 watts/min (T2D-nonHF) as previously described [11]. Blood pressure, heart rate, and ECG were measured at rest and repeatedly every second minute during the exercise test. The exercise echocardiography was performed immediately after maximal exercise.

### 6-minute hall walk test

The T2D-HF patients performed a 6-minute hall walk test after echocardiography. The test was carried out on a straight 50-meter indoor course. The test was applied in addition to the exercise test as it is considered to reflect daily life activity in HF patients [20]. It was not performed in the T2D-nonHF group as we would not expect the test to be compromised in patients without HF.

### Outcomes

Primary endpoints were defined as differences in LVEF and tissue-Doppler-derived measurements of systolic contractile function between hyper- and normoglycemia in T2D patients. Secondary endpoints were walking distance during a 6-minute hall walk test (in T2D-HF patients), peak exercise capacity, left ventricular function, and post-exercise tissue-Doppler-derived measures of systolic contractile function. The outcomes were measured consecutively and analyzed after completion of T2D patients with both preserved and reduced LVEF.

### Statistics

We applied the Kolmogorov normality test to test for Gaussian distribution and transformation was performed when appropriate. Non-paired t-test was used to test for differences between groups. Two-way ANOVA with repeated measurements was applied to test for differences between study arms (hyperglycemia vs. normoglycemia) and interaction of HF status (HF and nonHF). We applied post-hoc Student-Newman-Keuls analysis to test for an effect in each study group unless stated otherwise. Correlations were investigated by Pearson's test. Data are presented as mean±SD unless stated otherwise. P-values <0.05 were considered significant.

Power calculations revealed that by enrolling 20 patients in cross-over-design (with an expected drop out of 10%) we expected to be able to detect relative changes in tissue-Doppler-derived measurements of systolic contractile function (S'max, Vmax with SD app. 8% [21]) in the order of 6% and absolute changes in LVEF (SD app. 5% [22]) in the order of 4% with 80% statistical power and a significance level of 5%.

## Results

### Patients (Figure 2)

**Figure 2:**
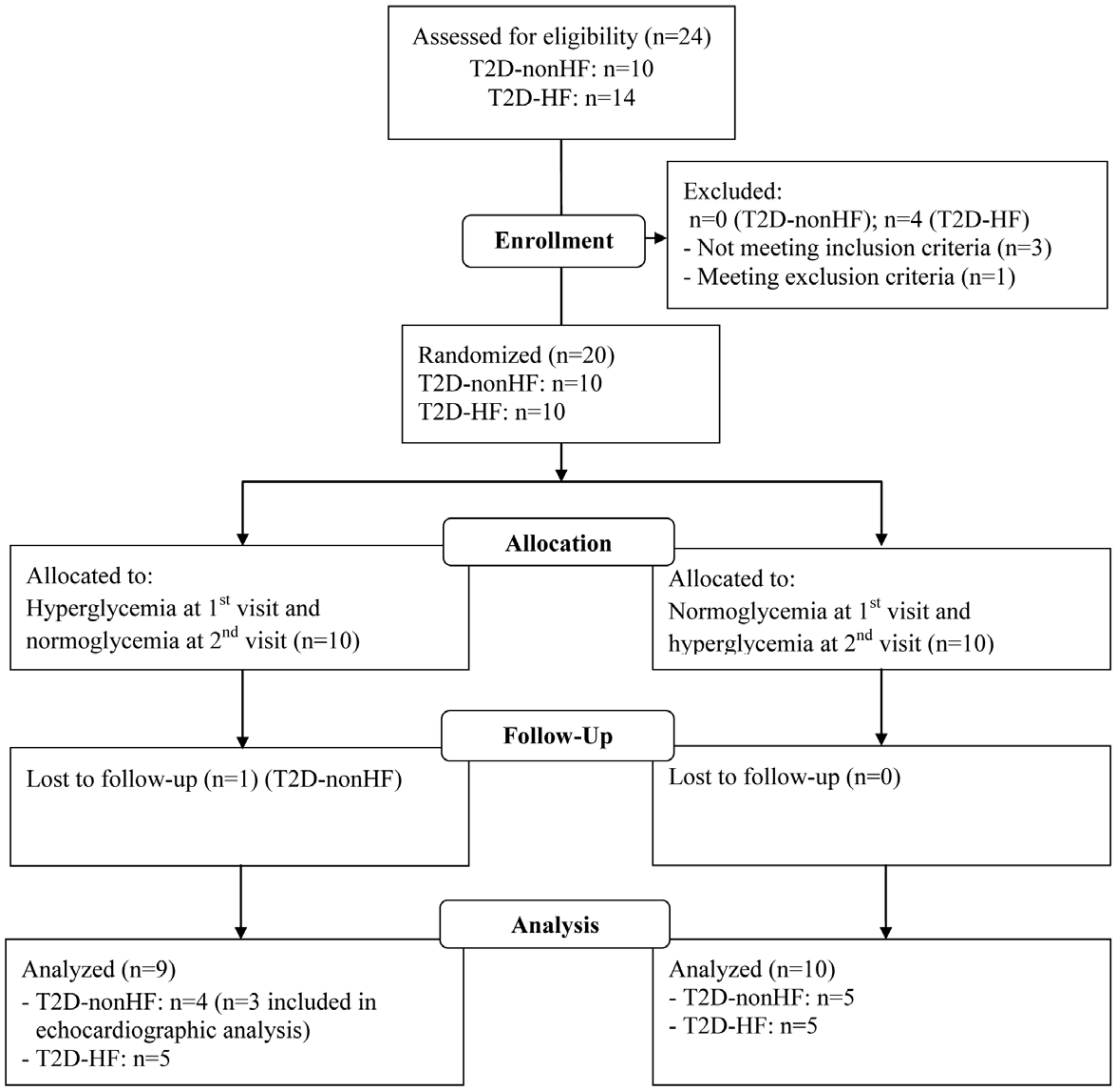
CONSORT diagram.

Based on our inclusion and exclusion criteria 20 (T2D-HF) out of 240 patients from our outpatient HF clinic and 19 out of 195 patients from our outpatient diabetes clinic (T2D-nonHF) were invited to participate. Of these 39 patients 24 agreed on screening and were assessed for eligibility (14 T2D-HF and 10 T2D-nonHF patients). After screening we enrolled 20 patients (10 T2D-HF and 10 T2D-nonHF patients). It was impossible to draw blood samples in one T2D-nonHF patient. Another T2D-nonHF patient declined echocardiography after the first visit (hyperglycemia) due to discomfort and was not included in the echocardiographic measurements. Thus, 19 patients completed normoglycemia and 18 patients completed hyperglycemia. Only patients completing both study arms are included in the present results (n = 18). No patients experienced hypoglycemia (lowest plasma glucose was 4.8 mM) and one patient (T2D-HF) experienced hyperglycemia >20 mM during the first two hours of the investigation. No symptoms of shortness of breath or dizziness were reported in either study arm. The T2D-nonHF patients had higher body weight, higher diastolic blood pressure and heart rate, and received less medication than the T2D-HF patients (Table 1). The volume infused was below 200 ml during normoglycemia. No infusion was administered during hyperglycemia, except during the first two hours to the patient who had plasma glucose >20 mM. The patients' weight did not differ between the study arms (T2D-HF: 90±13 kg (normoglycemia) vs. 90±14 kg (hyperglycemia), T2D-nonHF: 112±10 kg (normoglycemia) vs. 112±10 kg (hyperglycemia), p = 0.21 (hyper- vs. normoglycemia)) and no difference was found in plasma volume using the Beaumont formula [23].

**Table 1:** Baseline characteristics.

	T2D-HF (n = 10)	T2D-nonHF (n = 9)	p-value
Age (years)	67±7	62±5	0.09
Duration of diabetes (years)	17±10.5	10±3.7	0.07
Sex (male(n)/female(n))	9/1	8/1	
Atrial fibrilation (n)	2	0	
Neuropathy (monofilament test) (n)	2	3	
Nephropathy** (n)	2	1	
Retinopathy (n)	3	2	
LVEF (%)	36±6	60±3	<0.001
Systolic BP (mmHg)	123±19	136±10	0.09
Diastolic BP (mmHg)	74±11	86±10	0.03
Heart rate (bpm)	67±14	87±16	<0.01
eGFR (mL/min)*	72±17	89±19	0.05
HbA1c (%)	8.7±1.6	8.0±1.4	0.36
Weight (kg)	90±13	110±10	<0.01
BMI	30±9	33±4	0.47
Medication			
Insulin (units/day)	75±51	80±26	0.78
ACE-inhibitors (n)	10	4	
Betablockers (n)	9	1	
Spironolactone (n)	4	0	
Oral anticoagulation (n)	2	0	
Statins (n)	10	7	
Other antihypertensive drugs (n)	1	4	
Acetylsalicylic acid (n)	10	6	
Biguanide (n)	0	7	
Other antidiabetics (n)	1	1	
Loop diuretics (n)	7	0	

Values are mean±SD. (LVEF: left ventricular ejection fraction; BMI: body mass index; BP: blood pressure.

* eGFR: estimated GFR by MDRD formula.

** Nephropathy defined as microalbuminuria (albuminuria >30 mg/L)).

The insulin dosage was reduced by approximately 70% (T2D-HF: 60±31 IE vs. 20±11 IE, p<0.001; T2D-nonHF: 61±14 IE vs. 14±11 IE, p<0.001) in each study group during hyperglycemia 24 hours before the investigations as compared with normoglycemia. The reduction in insulin did not differ between study group (p = 0.97, test for interaction).

### Plasma glucose levels (Figure 3+Table 2)

**Figure 3:**
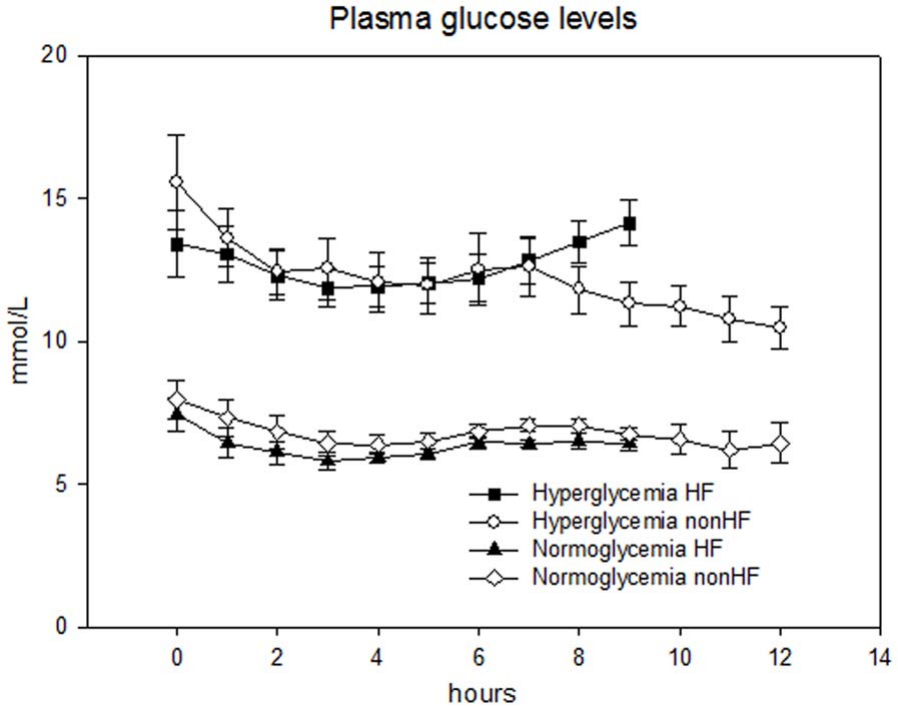
Glucose levels. Hourly glucose measurements in both study groups (T2D-HF and T2D-nonHF) during both interventions (hyperglycemia and normoglycemia). Glucose levels differed significant between hyperglycemia and normoglycemia (p<0.001, two-way ANOVA with repeated measurements) in both study groups (T2D-HF: p<0.001, T2D-nonHF: p<0.001). Neither during hyper- (p = 0.28) nor normoglycemia (0.24) did the two study populations differ with respect to glucose levels (bars indicating mean±SEM).

Plasma glucose levels differed significantly between hyper- and normoglycemia. The differences were significant at all hourly measurements performed before the investigations in each study group. The measured glucose levels did not differ between study groups neither during hyper- (p = 0.28) nor normoglycemia (p = 0.24). However, HF status had significant interaction on the glucose levels measured just prior to the investigations (p = 0.001) (table 2).

**Table 2:** Blood sample measurements.

	T2D-HF		T2D-nonHF		Two-way ANOVA
	Nor. (n = 10)	Hyp. (n = 10)	Nor. (n = 8)	Hyp. (n = 8)	p-value
P-glucose	6.5±0.8	14.1±2.6**	5.8±0.4	9.9±2.1**	<0.001 †
FFA (mmol/L)	0.51±0.23	0.75±0.24*	0.51±0.15	0.65±0.13	0.008
Insulin (pmol/L)	153±140	103±75	162±58	104±36	0.01
C-peptide (pmol/L)	617±393	1353±752**	401±274	1228±280**	<0.001
NT-proBNP (ng/L)	925±1100	946±1295	55±56	45±51	0.92

Blood sample measurements prior to the investigations. Values are mean±SD. Two-way ANOVA with repeated measurements was applied to test for difference between hyper- and normoglycemia and interaction (Nor: Normoglycemia. Hyp: Hyperglycemia).

** : p-value <0.01 between normoglycemia vs hyperglycemia (Student-Newman-Keuls post-hoc analysis).

* : p-value <0.001 between normoglycemia vs hyperglycemia (Student-Newman-Keuls post-hoc analysis).

† : There was significant interaction of HF on glucose levels between study groups (p = 0.001).

### Hormones and substrates (Table 2)

The circulating insulin levels were lower, whereas the FFA and C-peptide levels were higher during hyperglycemia than during normoglycemia. NT-proBNP (Table 2), catecholamines, cortisol, growth hormone, and IGF-1 (data not shown) did not differ between the study arms.

### Echocardiography (Table 3+Figure 4a+b)

**Figure 4:**
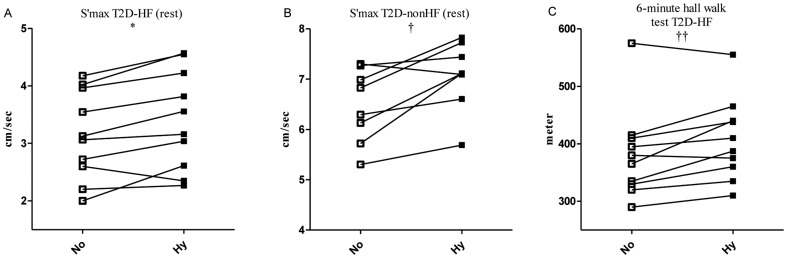
Myocardial contractile function and 6-minute hall walk test. Tissue-Doppler mitral plane longitudinal maximal velocity (S'max) in **A:** T2D-HF group and **B:** T2D-nonHF group during resting state. **C:** 6-minute hall walk test performed in the T2D-HF group (C). No: normoglycemia; Hy: hyperglycemia. * Difference (hyper- vs normoglycemia): 9%, p = 0.04. † Difference: (hyper- vs normoglycemia): 10%, p<0.001. †† Mean difference 26 meter corresponding to 7%, p = 0.02, paired t-test.

**Table 3:** Echocardiographic and exercise measurements.

	T2D-HF		T2D-nonHF		Two-way ANOVA
	Nor (n = 10)	Hyp (n = 10)	Nor (n = 8)	Hyp (n = 8)	p-value
**Resting data:**					
LVEF (%)	35±5	36±6	59±4	60±4	0.17
Vmax (cm/sec)	2.1±0.7	2.4±0.9†	3.6±0.4	4.0±0.3**	<0.001
S'max (cm/sec)	3.1±0.8	3.4±0.9†	6.5±0.7	7.1±0.7*	<0.001
Strain (%)	8.3±2.8	9.1±3.6	13.4±2.5	14.4±1.9	0.05
Strain rate (sec^−1^)	0.48±0.08	0.52±0.13	0.78±0.13	0.84±0.11	0.02
MAP (mmHg)	93±10	88±13	95±16	99±10	0.77
Heart rate (bpm)	66±17	63±14	89±17	90±12	0.66
**Exercise data:**					
EF (%)	39±6	39±8	64±7	62±5	0.59
Vmax (cm/sec)	3.0±1.4	3.2±1.8	5.3±0.9	5.5±1.4	0.17
Peak S'max (cm/sec)	4.0±1.2	4.4±1.7	9.3±1.9	9.7±2.6	0.049
Peak strain (%)	9.9±3.6	9.3±3.	14.0±2.0	14.8±1.8	0.93
Peak strain rate (sec^−1^)	0.64±0.19	0.67±0.27	0.96±0.15	1.06±0.24	0.13
Peak capacity (watt)	98±19	101±16	143±19	149±22	0.30
MAP (mmHg)	106±15	103±16	113±9	116±10	0.92
Heart rate (bpm)	107±22	108±22	125±20	125±18	0.68

Echocardiographic measurements and bicycle exercise test for each study group. Values are mean±SD. Two-way ANOVA with repeated measurements revealed no significant interaction. Underlined values indicate p<0.05.

* : p-value <0.001,

** : p-value <0.01,

† : p-value <0.05 between normoglycemia vs. hyperglycemia (Student-Newman-Keuls post-hoc analysis).

Nor: Normoglycemia. Hyp: Hyperglycemia. LVEF: left ventricular ejection fraction. S'max: longitudinal mitral plane maximal velocity. Vmax: Global myocardial maximal systolic tissue velocity.

A total of 212 myocardial segments were analyzed (116 in T2D-HF and 92 in T2D-nonHF). Eight segments could not be analyzed due to poor acoustic windows. Hyperglycemia was associated with an increase in the following parameters: Vmax (p<0.001), S'max (p<0.001), strain rate, (p = 0.02) and strain (near-significant: p = 0.05). Indices of increased myocardial systolic contractile function were significant in both T2D-HF (Vmax: 14%, p = 0.02; S'max: 10%, p = 0.04), and T2D-nonHF (Vmax: 12%, p<0.01; S'max: 9%, p<0.001) during hyperglycemia as opposed to normoglycemia. LVEF did not differ between normo- and hyperglycemia (p = 0.17). Neither did systolic duration (p = 0.33), diastolic duration (p = 0.60) nor diastolic function (E-wave velocity, A-wave velocity, E/A-ratio, e' velocity or E/e') between the study arms (data not shown). There was no interaction of HF status on our outcomes (hyper- vs. normoglycemia).

The glucose levels and S'max correlated inversely during hyperglycemia (r^2^ = 0.27, p = 0.03), whereas the increase in myocardial contractile function and glucose levels from normo- to hyperglycemia was not correlated (p = 0.60). Furthermore, there was no correlation between myocardial contractile function and FFA, insulin or C-peptide levels (data not shown).

### Post exercise echocardiography (Table 3)

Compared with normoglycemia, hyperglycemia increased post exercise mean S'max by app. 7% (4.0±1.2 cm/sec vs 4.4±1.7 cm/sec (T2D-HF) and 9.3±1.9 cm/sec vs 9.7±2.6 cm/sec (T2D-nonHF), p = 0.049)). There was no interaction of HF status on S'max post exercise. The functional longitudinal systolic (p = 0.36) and diastolic (p = 0.95) reserve indexes did not differ between the study arms.

### Hemodynamics (Table 3)

Neither mean arterial blood pressure (MAP) nor heart rate differed between normo- as opposed to hyperglycemia and we observed no differences in systolic (p = 0.77) or diastolic blood pressure (p = 0.61) between study arms.

### 6-minutes hall walk test (Figure 4c)

The walking distance rose from 382±79 meters to 408±71 meters (p = 0.02, paired t-test) during hyperglycemia as compared with normoglycemia in T2D-HF patients. This corresponds to a mean increase of 26 meters or 7%. No patients complained of knee or leg pain.

### Exercise testing (Table 3)

There were no differences in exercise capacity, MAP, heart rate, systolic (p = 0.41), or diastolic blood pressure (p = 0.70) between study arms. All T2D-nonHF ceased due to physical exhaustion whereas nine of the ten T2D-HF ceased due to knee or leg pain.

## Discussion

In the present studies, 9 to 12 hours of hyperglycemia induced by insulin discontinuation increased left ventricular contractile function in T2D patients irrespective of whether LVEF was preserved or reduced. Hyperglycemia was associated with a longer 6-minute hall walk distance in patients with T2D and HF.

To our knowledge, no previous studies have addressed the effects of short-term hyperglycemia by insulin discontinuation on left ventricular contractile and circulatory function at rest and exercise in T2D patients with and without HF. The present results argue against our hypothesis and were in part unexpected as deranged whole body metabolism is considered harmful in HF patients, particularly when HF coexists with diabetes [24].

The cardiovascular effects of reducing circulating FFA [11], [13] and modulating whole body and myocardial metabolism [12], [14], [25] have been inconsistent. This inconsistency may be rooted in differences between studies in terms of endpoints [12], [14], patient populations [13], [25], methods of intervention [12]–[14], [25], [26], unintended side effects [26], and the potential confounding effects of variable plasma glucose levels. Prior investigations have mainly applied hyperinsulinemia and kept plasma glucose at approximately 5 mM [12], [14], [25]. However, in the present studies, a rise in plasma glucose levels was accomplished by reducing insulin dosage by app. 70%. This increased plasma glucose by app. 100%, lowered insulin levels by app. 35%, and increased FFA by app. 35%. Hence, the resulting increase in myocardial contractile function supports that in the presence of circulating insulin, a short-term, ample glucose and FFA supply may have beneficial short-term effects. Catecholamines did not differ between normo- and hyperglycemia, and we observed no changes in systemic hemodynamics, which demonstrates the absence of any difference in vascular resistance. Weight and plasma volume were similar in both the study arms. This argues against any diuretic effect biasing our results.

We observed a negative correlation between glucose levels and myocardial contractile function during hyperglycemia. This we believe is due to the fact that T2D-nonHF had lower glucose levels and higher myocardial contractile function per se as opposed to T2D-HF patients in whom the glucose levels were highest and contractile function lowest.

In a previous study, we found no influence of circulating FFA and insulin on left ventricular contractile function in HF patients [14]. We therefore find it less likely that the increase in circulating FFA and the reduced insulin levels contributed considerably to the present findings. Hence, we suggest that a short-term rise in plasma glucose levels induced by insulin discontinuation can improve the cardiac contractile function in T2D patients with and without HF independently of other major metabolic changes. This effect may be ascribed to an increase in MGU because of the glucose mass effect [27], and a simultaneous reduction in FFA uptake and oxidation as described by Randle [28]. We have previously demonstrated that MGU rises steeply during normoglycemia in parallel with insulin levels up to approximately 100 pmol/l [29]. Above this level, the increase in MGU is small; and we therefore hypothesize that the combination of short-term hyperglycemia and the presence of circulating insulin of app. 160 mmol/L as found in the present studies could further increase MGU by the glucose mass effect [27] and increase the contractile function through metabolic effects. In addition, hyperglycemia increases intracellular calcium levels in cardiomyocytes independently of insulin. This could also have contributed to the present findings [30]. It is intriguing that a similar contractile response to hyperglycemia was observed in both patients with and without HF in spite of a larger increase in glucose levels in T2D-HF. This observation may be due to myocardial contractility increasing less in T2D-HF patients as compared toT2D-nonHF in response to hyperglycemia or due to a “saturation” of myocardial contractility in response to increasing glucose levels. Moreover, this finding also suggests that similar mechanisms are involved in the two patient categories.

A previous study reports that glucose levels acutely increased by glucose infusion in T2D patients with normal LVEF had no effect on global LV function [31]. However, in the present studies more sensitive measurements based on tissue-Doppler velocity unambiguously demonstrated that left ventricular contractile function was higher during hyperglycemia than during normoglycemia.

Whether hyperglycemia induced by insulin discontinuation affects the function and performance of other organs, i.e. the lungs or muscles, and thereby improves walking distance and thus causes the present difference cannot be excluded [32]. However, we chose to add both bicycle exercise test and 6-minutes hall walk test in the T2D-HF group as the latter is believed to reflect daily activity as opposed to laboratory exercise capacity [20]. Thus, these tests contribute with different information. This, and the fact that nearly all TD2-HF patients ceased the exercise test due to knee/leg pain, may explain that the exercise capacity was not affected whereas the hall walk distance increased during hyperglycemia in the T2D-HF patients.

LVEF was unchanged. However, the effect on S'max may have clinical relevance. S'max has been shown to be an independent predictor of mortality in the general population [33]. The present studies therefore support that short-term ample availability of glucose and FFA caused by insulin discontinuation does not depress LVEF and may, as previously hypothesized [34], have beneficial short-term effects in patients with T2D and HF. Our present studies show that the glycemic level in T2D patients biases tissue-Doppler-derived indices of myocardial contractile function and should therefore be taken into account during the interpretation of such measurements in future clinical trials.

It should be emphasized that the present studies address short-term diabetic dysregulation, whereas long-term dysregulation involves different pathophysiological processes [28]. Nevertheless, previous long-term studies indicate that achieving tight glycemic control in diabetic patients does not significantly improve cardiac function. It may either have no beneficial effect or even paradoxically decrease LVEF in T2D-nonHF patients with coronary heart disease [35] and increase death from cardiovascular disease although the number of microvascular complications is reduced [9], [36]. The present results are in accordance with these previous long-term findings and argue against the beneficial effects of tight glycemic control in T2D patients during an acute setting if increased myocardial contractile function is vital. However, based on the present studies we cannot conclude whether normoglycemia is detrimental or hyperglycemia is beneficial. This needs to be addressed in future trials.

The design of the studies resulted in variable glycemic levels during hyperglycemia as opposed to the well-controlled normoglycemic situation. Nevertheless, we find the design to be of clinical relevance as these opposite scenarios (hyperglycemia vs. normoglycemia) correspond to a lenient or tight glycemic strategy during hospitalization of T2D patients with reduced left ventricular contractile function. However, whether the long-term effect of tight as opposed to lenient glucose control on LVEF or contractile function is beneficial in T2D-HF patients requires a long-term randomized study.

## Study Limitations

The possibility of a type 2 statistical error on LVEF must be considered. We used a cross-over design and sensitive echocardiographic techniques with two-way ANOVA with repeated measurements to address myocardial contractile function. This approach eliminated inter-patient and minimized intra-patient variation between study arms. Nevertheless, the present investigation is composed of two minor trials and thus it should be interpreted with caution. It is possible that the echocardiographic techniques used to assess volumes were insensitive to subtle changes in LVEF. However, our data consistently demonstrated that myocardial systolic contractile function was higher during hyperglycemia than during normoglycemia in T2D patients irrespective of whether the patients had normal or reduced LVEF.

Measurements of left ventricular contractile function were performed on two different versions of the Vivid GE echo-machines. To our knowledge no evidence suggests that this could bias the present results. Moreover, each patient was examined using the same echo-machine at both visits, the applied technics were similar, and all analyses were performed in the same matter and on the same platform (EchoPac 9).

We report on several correlations between myocardial contractile function, substrates and hormones. However, the present studies were designed to evaluate on the effect of overall metabolic derangement occurring during hyperglycemia due to insulin discontinuation. Thus, these correlations should be interpreted with caution.

Euinsulinemic hyperglycemia at glucose levels of 12–13 mM increases blood pressure and lowers heart rate and cardiac index in healthy subjects [37]. Such changes could potentially have affected our results. However, this seems unlikely since we observed no differences in heart rate or blood pressure between the study arms. Hyperglycemia could have caused diuresis and fluid depletion and thus have affected our measurements due to preload differences between the study arms. However, neither weight, plasma volume, E-wave nor e' velocity differed between the study arms which indicates that there were no differences in preload [38].

None of the T2D-HF patients as opposed to most of the T2D-nonHF patients were on metformin (biguanide) treatment. Metformin has been shown to have beneficial effects on left ventricular function in experimental models [39]. Whether this could have biased our results are unknown. We sought to lower the risk of this potential bias by pausing metformin 2 days prior to the investigations. Nevertheless, it cannot be excluded that the long-term effects of metformin affected our results.

C-peptide differed between the study arms. It has been shown that C-peptide increases LVEF and myocardial perfusion in patients with type 1 diabetes and normal LVEF. Nevertheless, increased C-peptide levels had no vascular effects during preserved endogenous C-peptide production [40], [41]. Since all patients in the present studies had endogenous C-peptide production in both study arms, we find it unlikely that different C-peptide levels have biased our results.

Only 2 females were included. We cannot exclude that acute insulin discontinuation and hyperglycemia have different effects in males and females. However, at present, no study supports that there are any gender difference in myocardial contractile function in response to short-term hyperglycemia.

We did not apply the hall walk test in the T2D-nonHF group as we did not expect it to be compromised. However, we cannot exclude that hyperglycemia as opposed to normoglycemia would have caused an effect on this low-grade exercise test in T2D-nonHF patients. It should be emphasized that the T2D-HF patients primarily ceased the exercise test due to knee/leg pain and not due to dyspnea or physical exhaustion. Therefore, we believe the 6-MWT to be a better test of exercise in the present T2D-HF patients even though it is a low-grade exercise test reflecting daily life activity [20]. However, these results should be interpreted with caution as the test was not applied in both study groups.

The T2D-nonHF group spent 3 hours more in each study arm as opposed to the T2D-HF group. Thus, we cannot rule out that the effect of hyperglycemia as opposed to normoglycemia may take longer to occur in the T2D-nonHF group. However, we believe the actual “interventions” were considerably longer than 9 and 12 hours as insulin dosage was reduced by app. 50% on the day of admittance and glucose levels already differed at time of the first glucose measurement at 12.00 midnight (figure 3).

We did not measure MGU. However, the feasibility of myocardial FDG-PET is known to be low if hyperinsulinemic clamp is not applied in T2D patients. Since we intended to investigate hyperglycemia as a result of insulin discontinuation as opposed to normoglycemia, we chose not to use FGD-PET in the present studies.

## Conclusions

Acute hyperglycemia induced by short-term insulin discontinuation surprisingly increases tissue-Doppler-derived indices of left ventricular contractile function in insulin-treated T2D patients with and without heart failure. Furthermore, acute hyperglycemia had no negative effects on LVEF or exercise capacity, and hyperglycemia was associated with a slight prolongation of walking distance by 6-minute hall walk test in T2D-HF patients. The exact mechanism of these findings and their potential clinical impact require further studies.

## Supporting Information

Protocol S1 (DOC)

Checklist S1 (DOC)
