# Supplementary material for: Effect of Acute Hyperglycemia on Left Ventricular Contractile Function in Diabetic Patients with and without Heart Failure: Two Randomized Cross-Over Studies
Source: PLoS One. 2013 Jan 8;8(1):e53247. doi: 10.1371/journal.pone.0053247 (PMC3540097; doi:10.1371/journal.pone.0053247)
Supplement: Protocol S1 — (DOC) [file pone.0053247.s001.doc]

***Projekt protokol***

***Metabolisk substrat modulering hos type 2 diabetikere med hjertesvigt: Effekt af hyperglykæmi på venstre ventrikelfunktion og arbejdskapacitet.***

***Baggrund***

Patienter med diabetes har en væsentligt øget risiko for at udvikle hjerteinsufficiens sammenlignet med ikke-diabetikere(1-3), og diabetikere med hjerteinsufficiens har en markant øget morbiditet og mortalitet (4;5) med en 1-års mortalitet i danske undersøgelser på 31%(6). Risikoen for at diabetikere udvikler hjerteinsufficiens er korreleret til dårlig metabolisk kontrol, idet risikoen for at udvikle hjerteinsufficiens stiger med 16% for hver 1% stigning i HbA1c(7). Patienternes metaboliske tilstand er kendetegnet ved høje niveauer af cirkulerende frie fedtsyrer (FFA), insulin og glukose(8;9), men det er uafklaret hvordan disse metaboliske abnormiteter bidrager til progression af hjertesvigtlidelsen. Høje FFA-niveauer kan medvirke til mindre effektiv energiproduktion i hjertets muskulatur ved hjerteinsufficiens. Yderligere er abnorm metabolisme i tværstribet muskulatur også vist at kunne bidrage til sygdomsprogression og dårlig prognose (10-12) Effekten af glukoseniveauerne på myokardiets kontraktilitet hos diabetikere er uklar(13;14) og det er uvist, hvorvidt akut diabetisk dysregulation med hyperglykæmi hos insulin behandlede type 2 diabetikere med og uden hjertesvigt påvirker hjertepumpefunktionen i hvile og under fysisk arbejde.

***Hypotese:***

Akut diabetisk dysregulation med hyperglykæmi påvirker hjertepumpefunktionen hos diabetikere med kronisk hjertesvigt.

***Formål:***

At undersøge hvorledes korterevarende ændringer i blodglukoseniveauerne påvirker hjertefunktion og arbejdskapacitet hos patienter med hjertesvigt.

## Deltagere

Inklusionskriterier: 13 patienter med insulinbehandlet type 2 diabetes og nedsat hjertepumpefunktion (ejection fraction <45%) Alder 30-80 år.

Eksklusionskriterier: Kendt nyreinsufficiens (Serum creatinin > 220 mmol/L, leverinsufficiens (ALAT > 3x normal området), tidligere apoplexi med tydelige invaliderende fysiske/psykiske sequlae, skizofreni, eller andre svære, invaliderende, konkurrerende lidelser og graviditet. Testdeltagere kan efter inklusion ekskluderes såfremt samtykket trækkes tilbage eller hvis testdeltageren udvikler svær, invaliderende, konkurrende lidelse. Projektet kan som helhed afbrydes hvis der er kausalitet mellem lidelsernes opståen og deltagelse i projektet.

## Design

Efter informeret samtykke og inklusion foretages indledende undersøgelse med ekkokardiografi, afprøvende arbejdstest og non-invasiv cardiac output måling (screening).

Dernæst planlægges 2 undersøgelsesdage i et randomiseret cross-over design. På den ene dag justeres blodsukkerniveauerne moderat hyperglykæmisk (mellem 16 og 18 mmol/L) og på den anden undersøgelsesdag justeres blodsukkerniveauerne euglykæmisk (mellem 6 og 8 mmol/L). Hver undersøgelsessituation udføres med minimum 2 ugers interval og per oralt antidiabetikum seponeres 2 døgn før hver undersøgelsesdag.

I den hyperglykæmiske situation møder testdeltageren på kardiologisk afdeling (dag 0) kl. 22.00 dagen før undersøgelsdagen. Vanlig insulinbehandling pauseres på dag 0 og der lægges en individuel plan for indgivning af insulin og testdeltageren instrueres heri. Der stilles mod at testdeltageren ca. får 60% af sin vanlige insulin. Der måles blodsukker 4 gange i løbet af dagen og ved blodsukkerniveau (BS) > 20 mmol/L gives ekstra 4 IE (internationale enheder) hurtigt virkende insulin mens der ved BS > 30 mmol/ gives ekstra 6 IE. Testdeltageren overnatter i afdelingen og den følgende dag (dag 1) gennemføres undersøgelserne. Metabolisk/hormonel profil (se blodprøver) tages kl. 8.00 på dag 1. Efterfølgende udføres Ekkokardiografi, arbejdstest, non-invasiv cardiac output måling og gangtest.

I den euglykæmiske situation instrueres testdeltagerne i at tage en individuel mængde insulin. Såfremt seneste HbA1c viser udtalt habituel dysregulation (HbA1c >0,09) vil deltagerne blive anmodet om at tage op til 25% ekstra insulin i løbet af dagen. Alle deltagere vil blive anmodet om at måle deres blodsukker 4 gange i løbet af dagen. De skal efterfølgende møde på kardiologisk afdeling kl. 22.00 (dag 0) og overnatter i afdelingen. Den følgende dag (dag 1) gennemføres samme undersøgelser som under den hyperglykæmiske situation.

I løbet af natten mellem dag 0 og dag 1 justeres sukkerniveauerne med intravenøs insulininfusion og om nødvendigt med supplering af glukoseinfusion stilende mod et blodsukkerniveau mellem 6 og 8 mmol/L i den ene situation og mellem 16 og 18 mmol/L i den anden situation.

Tidslinie:


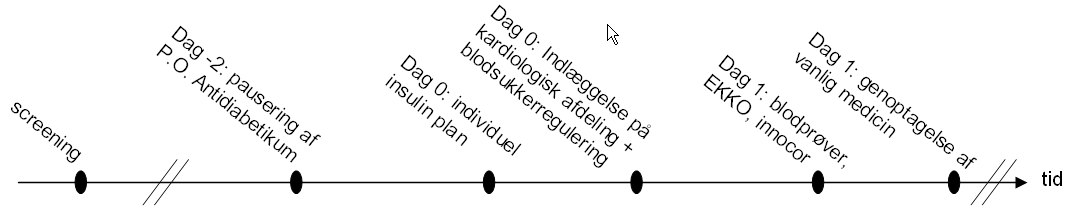


Figur 1. - Tidslinie: Perioden mellem dobbeltstregerne gentages hos hver testdeltager med henholdsvis hyper- og euglykæmi i randomiseret rækkefølge med minimum 2 ugers interval. Testdeltagerne indlægges i begge situation kl. 22.00.

***Effektparametre***

Primære

- Global venstre ventrikel funktion (i hvile og efter exercise test)
- Cardiac output i hvile og under maksimal anstrengelse
- Arbejdskapacitet og maksimal iltoptagelse
- Metabolisk/hormonel profil (se blodprøver)

Sekundære

- Regional venstre ventrikel funktion (tissue-Doppler i hvile og efter exercice test)

## Procedurer og Metoder

*Ekkokardiografi:* GE VIVID 7 systemet benyttes. De følgende parametre registreres: global uddrivningsfraktion (3D-ekko)(15), regional wall motion score, (16 segment model), forkortningsfraktion, M-mode af venstre atrium, pulset Doppler i mitralannulus, vævs-Doppler undersøgelse og speckle-tracking(16), diastolisk funktion (early diastolic myocardial velocity, E/A ratio, EdecT, IVRT, E/e’-ratio). Alle målinger beregnes som gennemsnit af 3-5 konsekutive hjerteslag. Der udføres målinger i hvile og efter maksimal anstrengelse.

*Arbejdstestning:* Arbejdstestning med cykelergometer under kontinuerlig EKG monitorering foretages på alle patienter. Belastningen øges 10W/minut. Hjertefrekvens, blodtryk, anaerob tærskel, maksimal iltoptagelse, non-invasivt cardiac output, maksimal arbejdskapacitet registreres.

*Non-invasivt Cardiac output:* INNOCOR apparaturet anvendes*.* Hvert tredje minut under arbejdstesten udføres genåndingstest hvor patienten i et lukket system indånder en blanding af atmosfærisk luft, ilt, lattergas og sulfur hexafluorid. Ved Fick´s princip kan lungernes gennemblødning og dermed Cardiac output beregnes. Der opnås endvidere en måling af maksimal iltoptagelse.

*6 minutters gangtest:* Udføres i.h.t. instruks på afd. B.

Blodtryk, puls, vægt registreres i hver undersøgelses situation før blodprøvetagning.

**Metoder - Generelle**

*Blodprøver (kl.89.00 dag 1)*: Blodprøver vil enten blive analyseret i forlængelse af blodprøvetagningen eller blive centrifugeret og afpipetteret og opbevaret ved -80 grader celcius mhp. analyse ved afslutning af projektet Der vil blive analyseret: catacholaminer, cortisol, frie fede syre, c-peptid, glucagon , glukose, NT-proBNP, hæmoglobin, hæmatokrit, albumin, creatinin, natrium, kalium, carbamid, alaninaminotransferase, adiponectin, TSH, amylin, ghrelin og IGF-1.

## Statistik

Parret t-test af deltaværdier (differencen mellem hyper- og euglykæmi) anvendes for at sammenligne ekkokardiografiske og biokemiske parametre samt arbejdskapacitet og gangdistance.

Alle værdier rapporteres som gennemsnit±SD (hvis normalfordelt) eller median[25-75 percentil]. En p-værdi <0.05 anses for signifikant.

Trevejs-ANOVA anvendes ved sammenligning af blodprøve-profiler (patient, hyper-/euglykæmi, tid).

Styrkeberegning:

n = 2 x *f*(*α,β*) x $\left(\frac{\sigma}{\delta}\right)^{2}$.

*α* er risikoen for type 1 fejl, *β* er risikoen for type 2 fejl, *σ* er standarddeviation og *δ* er den forskel vi ønsker at kunne detektere. Der forventes drop-out på 25%, dvs. 2-3 personer af de 13 inkluderede. Hvorfor ”n” bliver 10-11.

*Ekkokardiografi*: I reproducerbarheds-undersøgelser findes SD på global EF bestemt ved 3D-ekkokardiografi at være ca. 5 %. Hvis *α* er 5 % og *β* er 20 % er *f* 7.9. Hvis vi ønsker at finde forskelle i EF på ca. 6,3 %, bliver n = 2 x 7.9 x (5/7)^2^ = 10.

*Arbejdskapacitet*: I undersøgelser findes SD på 10 % mht. exercise tid og maksimal iltoptagelse. Hvis vi ønsker at kunne finde forskelle i arbejdskapacitet på ca. 12,6 %, bliver n = 2 x 7.9 x (10/14)^2^ = 10.

Non-invasiv cardiac output måling: Der foreligger ikke reproducerbarhedsundersøgelser under fysisk belastning men i hvile undersøgelser findes variansen på 7%(17), hvis vi ønsker at finde forskelle i cardiac output på ca. 3,3% bliver n=2 x 7,9 x 7/4^2^ = 10.

### Etik - Generelt

Forsøgene udføres i overensstemmelse med protokollen og gældende lovgivning. Undersøgelsernes påbegyndelse og gennemførelse forudsætter godkendelse fra Videnskabsetisk komité. Undersøgelserne udføres desuden i overensstemmelse med Helsinki Deklaration II’s krav til humane kliniske undersøgelser. Data fra både den euglykæmiske og hyperglykæmiske situation vil, uafhængigt af resultaterne, blive offentligtgjort. Dvs. at både positive og negative resultater offentliggøres. Projektet vil blive anmeldt til Datatilsynet. Da blodprøverne ikke analyseres umiddelbart i relation til at de udtages, oprettes en biobank, der ligeledes meldes til datatilsynet. Biobanken vil blive nedlagt ved terminering af projektet og eventuelt oveskydende blod/serum/plasma destrueret.

### Rekruttering og retningslinjer for skriftlig og mundtlig information

Forsøgsdeltagerne rekrutteres fra hjertesvigtklinikkerne på Kardiologisk afdeling B, Skejby Sygehus, Kardiologisk afdeling, Vejle sygehus samt vestdansk hjertedatabase og fra diabetes ambulatoriet, Medicinsk afdeling M, Århus sygehus. De patienter, der opfylder inklusionskriterierne kontaktes pr. brev, indeholdende skriftligt informationsmateriale. I det skriftlige informationsmateriale vil patienten blive informeret om muligheden for at medbringe en bisidder til en mundtlig informationssamtale. Såfremt den potentielle forsøgsperson ønsker at deltage i forsøget eller blot ønsker at vide mere udfyldes en blanket, der sendes til investigator, hvorefter den mulige forsøgsperson kontaktes telefonisk af investigator og der vil her blive aftalt tid og sted for mundtlig information. Den mundtlige information om undersøgelsen vil foregå i enerum og vil blive givet af læge Roni Nielsen eller afdelingslæge Henrik Wiggers. Der vil være mulighed for at stille spørgsmål og for at frasige sig viden om egen helbredstilstand. Folderen ”Dine rettigheder som forsøgsperson i et biomedicinsk forskningsprojekt” udgivet af Den Centrale Videnskabsetiske Komité, vil blive udleveret. Det vil blive understreget, at deltagelsen er frivillig og at tilsagn om deltagelse derfor kan trækkes tilbage når som helst, uden at dette på nogen måde vil påvirke læge-patient forholdet. Efter informationssamtalen vil der være betænkningstid inden indhentelse af skriftligt samtykke. Samtykkeerklæringen underskrives af forsøgspersonen og sendes til investigator.

Forsøgspersonerne har mulighed for at få yderligere oplysninger om projektet ved at ringe til den projektansvarlige.

Den mundtlige og skriftlige information gives i overensstemmelse med Forskningsstyrelsens Vejledning om information og samtykke ved inddragelse af forsøgspersoner i biomedicinske forskningsprojekter.

### Forudsigelige risici og ulemper

Blodprøvetagning er forbundet med lette smerter og der er en beskeden risiko for infektion og blodansamling ved indstiksstedet. Forsøgspersonerne vil blive orienteret om tegnene på infektion og instrueret i at kontakte en af de projektansvarlige læger og komme til vurdering på sygehuset. Der anvendes almindelig steril teknik. Det samlede blodtab andrager ca. 50 ml i hver undersøgelsessituation, hvilket ikke forventes at give symptomer og medføre utilpashed. I forbindelse med undersøgelserne på afdeling B vil der blive lagt intravenøs adgang i 2 perifere vener, dette kan være forbundet med let smerte. Der er i meget sjældne tilfælde beskrevet infektion efter anlæggelse af sådanne katetre.

Ultralydsscanning af hjertet foregår med gelé på huden og er smerte- og komplikationsfrit.

Arbejdstestning kan udløse brystsmerter hos patienter med angina pectoris og kan i sjældne tilfælde udløse farlige rytmeforstyrrelser. Arbejdstestning er en rutinemæssig undersøgelse på alle Kardiologiske afdelinger . Alvorlige komplikationer ved arbejdstest angives til <1/10000(18).

Under arbejdstesten udføres non-invasiv cardiac outputmåling ved genånding i pose. Denne procedure er ufarlig for patienten.

Alle nævnte metoder er velkendte i forsøgslaboratoriet og af de projektansvarlige og vurderes at være absolut acceptable i relation til det overordnede formål.

Fornemmelsen af hyperglykæmi er individuel, men det er velkendt at det kan fremkalde øget vandladning, tørst og træthed.

Risikoen for hypoglykæmi under forsøget betragtes som minimal da testpersonernes blodsukker følges kontinuerligt (hver time) mens de opholder sig på kardiologisk afdeling B. Infusion af insulin og glukose vil blive reguleret i henhold til blodsukkeret.

***Fordele ved at deltage i undersøgelsen***

Forsøgspersonen kan efter projektets afslutning få oplyst resultater af egne undersøgelser og svar på blodprøver samt kardiel status. Forsøgspersonen vil bidrage til ny vigtig viden, som han eller hun i sidste ende muligvis selv vil komme til at drage fordel af.

***Kompensation***

Deltagerne i undersøgelsen, vil på individuel basis modtage godtgørelse for tabt arbejdsfortjeneste, samt refusion af udgifter til transport etc. Godtgørelsen for tabt arbejdsfortjeneste er skattepligtig.

***Opbevaring og behandling af data***

Data beskyttes efter lov om behandling af personoplysninger og sundhedsloven. Data vil blive behandlet strengt fortroligt og vil kun blive publiceret i anonymiseret form. Data arkiveres på Hjertemedicinsk forskningsafdeling B, Skejby sygehus. Data skal arkiveres i 15 år efter undersøgelsens afslutning.

***Samarbejdspartnere***

Professor, dr.med Hans Erik Bøtker, afdelingslæge dr.med Henrik Wiggers, Læge Mads Halbirk, (Kardiologisk afd. B, Århus Universitetshospital, Skejby Sygehus), Professor dr.med Torsten Toftegaard.

Professor, dr.med Niels Møller (Medicinsk Forskningslaboratorium/Klinisk Institut, Århus UniversitetÅrhus Sygehus), Overlæge dr.med. Helene Nørrelund (Medicinsk afdeling, Viborg Sygehus)

Overlæge Ph.D. Flemming Hald Steffensen (Kardiologisk afdeling Vejle sygehus).

## Arbejdsfordeling

Læge Roni Nielsen vil stå for rekrutteringen af patienterne, indberetning og kommunikationen til de respektive etiske instanser. Samtlige undersøgelser udføres af Læge Roni Nielsen. Projektet vil blive en del af Læge Roni Ranghøj Nielsen’s Ph.d. projekt.

De kardiologiske undersøgelser supervisers og vejledes af Afdelingslæge, dr.med, Ph.d. Henrik Wiggers (Kardiologisk afd. B, Skejby sygehus). Den diabetiske regulering superviseres og vejledes af overlæge, dr.med, Ph.d. Helene Nørrelund (medicinsk afdeling, Viborg sygehus) og overlæge Dr. Med Niels Møller (Medicinsk afdeling M, Århus sygehus) med assistance fra læge ph.d. studerende Ulla Kampmann og medicinstuderende tilknyttet medicinsk forskningslaboratorium, Århus Sygehus, Nørrebrogade.

Professor, dr.med, Ph.d. Hans Erik Bøtker (Hjertemedicinsk afd. B Skejby sygehus) vil blive hovedvejleder. Hans Erik Bøtker vil derfor deltage i alle aspekter af projektet.

Læge Mads Halbirk (hjertemedicinsk forskningsafdeling, skejby sygehus) og Overlæge Flemming Hald Steffensen (Kardiologisk afdeling, Vejle sygehus) vil, sammen med ovenfor nævnte deltagere, assisterer i fortolkning af data

## Finansiering og Forsikring

Deltagerne er dækket i overensstemmelse med ”lov om klage- og erstatningsadgang inden for Sundhedsvæsnet”. Der vil blive søgt støtte fra Hjerteforeningen, Diabetesforeningen, Den sundhedsvidenskabelige forskningsfond Region midtjylland, Sundhedsvidenskabeligt fakultet Århus universitet.

## Publikationer

Projektet er en forlængelse af et allerede igangsat projekt omhandlende akut induceret hyperglykæmi hos insulinbehandlede type 2 diabetikere uden hjertesvigt. Resultaterne fra projekterne vil blive sammenholdt og forventes at danne baggrund for 1-2 originalartikel, der vil blive publiceret i internationale tidsskrifter.

## Tidsplan

Første patient planlægges til maj 2009

Forventet afsluttet: februar 2011

## Sted

Kardiologisk forskningsafsnit B, Skejby sygehus

***Referencer***

1. Haffner SM, Lehto S, Ronnemaa T, Pyorala K, Laakso M (1998) Mortality from coronary heart disease in subjects with type 2 diabetes and in nondiabetic subjects with and without prior myocardial infarction. N.Engl.J Med 339: 229-234

2. Woodfield MD, Lundergan MD, Reiner MD, Greenhouse P (1996) Angiographic Findings and Outcome in Diabetic Patients Treated With Thrombolytic Therapy for Acute Myocardial Infarction: The GUSTO-I Experience. Journal of the American College of Cardiology 28: 1661-1669

3. Malmberg K, Ryden L, Hamsten A, Herlitz J, Waldenstrom A, Wedel H (1997) Mortality prediction in diabetic patients with myocardial infarction: experiences from the DIGAMI study. Cardiovascular Research 34: 248-253

4. Malmberg K, Norhammar A, Wedel H, Ryden L (1999) Glycometabolic State at Admission: Important Risk Marker of Mortality in Conventionally Treated Patients With Diabetes Mellitus and Acute Myocardial Infarction : Long-Term Results From the Diabetes and Insulin-Glucose Infusion in Acute Myocardial Infarction (DIGAMI) Study. Circulation 99: 2626-2632

5. Shindler DM, Kostis JB, Yusuf S, et al (1996) Diabetes mellitus, a predictor of morbidity and mortality in the studies of left ventricular dysfunction (SOLVD) trials and registry. The American Journal of Cardiology 77: 1017-1020

6. Gustafsson I, Brendorp B, Seibaek M, et al (2004) Influence of diabetes and diabetes-gender interaction on the risk of death in patients hospitalized with congestive heart failure. Journal of the American College of Cardiology 43: 771-777

7. Stratton IM, Adler AI, Neil HA, et al (2000) Association of glycaemia with macrovascular and microvascular complications of type 2 diabetes (UKPDS 35): prospective observational study. BMJ 321: 405-412

8. Boden G (2003) Effects of Free Fatty Acids (FFA) on Glucose Metabolism: Significance for Insulin Resistance and Type 2 Diabetes. Experimental and Clinical Endocrinology &amp; Diabetes 121-124

9. Norrelund H, Wiggers H, HALBIRK M, et al (2006) Abnormalities of whole body protein turnover, muscle metabolism and levels of metabolic hormones in patients with chronic heart failure. Journal of Internal Medicine 260: 11-21

10. Anker SD, Ponikowski P, Varney S, et al (1997) Wasting as independent risk factor for mortality in chronic heart failure. The Lancet 349: 1050-1053

11. Murray AJ, Anderson RE, Watson GC, Radda GK, Clarke K (4 A.D.) Uncoupling proteins in human heart. The Lancet 364: 1786-1788

12. Opie LH The metabolic vicious cycle in heart failure. The Lancet 364: 1733-1734

13. George AKM, Shih AM, Regan TJM (1996) Effect of Acute Ketoacidosis on the Myocardium in Diabetes. [Article]. American Journal of the Medical Sciences 311: 61-64

14. Wiggers H, Norrelund H, Nielsen SS, et al (2005) Influence of insulin and free fatty acids on contractile function in patients with chronically stunned and hibernating myocardium. Am J Physiol Heart Circ Physiol 289: H938-H946

15. Kim W, Sogaard P, Kristensen B, Egeblad H (2001) Measurement of left ventricular volumes by 3-dimensional echocardiography with tissue harmonic imaging: A comparison with magnetic resonance imaging. J Am Soc Echocardiogr 14: 169-179

16. Teske AJ, De Boeck BW, Melman PG, Sieswerda GT, Doevendans PA, Cramer MJ (2007) Echocardiographic quantification of myocardial function using tissue deformation imaging, a guide to image acquisition and analysis using tissue Doppler and speckle tracking. Cardiovasc.Ultrasound 5: 27

17. Hunt BE, Davy KP, Seals DR (1997) Reproducibility of a semiautomated acetylene rebreathing technique for measuring cardiac output in humans at rest. Clinical Physiology 17: 599-607

18. Gibbons L, Blair SN, Kohl HW, Cooper K (1989) The safety of maximal exercise testing
65. Circulation 80: 846-852

## Delprojekt 2: Formål

At karakterisere mekanismer bag de metaboliske forstyrrelser hos akut dysregulerede danske type-2-diabetikere i form af insulin- og cytokin signalveje og ”proteomics” i fedt og muskel biopsier. Herudover ønsker vi at undersøge effekten af akut hyperglykæmi på carbamid syntese hastigheden (et udtryk for proteinnedbrydningen), aminosyreomsætningen, glucoseomsætningen og lipidomsætningen.

Desuden vil vi undersøge om akut dysregulation påvirker hjertes pumpefunktion.

## Deltagere

10 patienter med insulinbehandlet type 2 diabetes.

Inklusionskriterier:

- Alder 40-75 år.
- BMI mellem 22 og 35.

Eksklusionskriterier:

- Svære, invaliderende, konkurrerende lidelser

Forsøgspersoner vil udgå af undersøgelsen, hvis:

- De selv ønsker det
- Forsøgsdeltager udvikler svær, invaliderende, konkurrerende lidelse

## Design

Efter informeret samtykke og inklusion skal hver forsøgsperson gennemgå en indledende undersøgelse med ekkokardiografi, afprøvende arbejdstest og non-invasiv cardiac output måling. Dernæst planlægges 2 undersøgelsesdage i et cross-over design. På begge undersøgelsesdage møder patienten på afdelingen kl. 22 aftenen før undersøgelsen. Vanlig insulinbehandling seponeres om morgenen på indlæggelsesdagen. For hver patient, der er i behandling med insulin lægges en individuel plan for indgivning af hurtigtvirkende insulin de sidste 24 timer op til undersøgelsesdagen. Der anlægges en venflon i en antecubital vene til blodprøvetagning og en venflon i modsidige håndrygsvene til infusioner. Patienterne skal overnatte på laboratoriet og skal faste fra midnat. Der gives intravenøs insulininfusion stilende mod et blodsukker niveau mellem 5 og 7 i den ene situation og mellem 18 og 20 i den anden situation.

Ved undersøgelsesdagens begyndelse, dvs. kl. 0800 påbegyndes indgivelse af carbamidtracer. Kl. 0900 indgives phenylalanin traceren, kl. 1000 indgives glucosetraceren og kl. 1100 indgives palmitattraceren. Kl. 1200 stoppes de metaboliske forsøg, hvorefter de cardiologiske undersøgelser påbegyndes. Ca. en time efter undersøgelsesdagens begyndelse tages muskel- og fedtbiopsierne. Hver undersøgelsessituation udføres med 2-4 ugers interval. Raske kontrol personer undersøges i fastende basaltilstand, gennemgår kun 1 undersøgelsesdag og vil ikke gennemgå de kardiologiske undersøgelser.

Effektparametre

Primære

- Global venstre ventrikel funktion (i hvile og efter exercise test)
- Arbejdstest
- Metabolisk/hormonel profil (se blodprøver)
- Biopsi analyser (insulin og cytokin signalveje, Proteomanalyser)
- Metaboliske analyser

Sekundære

- Regional venstre ventrikel funktion (tissue-Doppler i hvile og efter exercice test)

## Procedurer og Metoder

Blodprøver: Ghrelin, GH, FFA, insulin, C-peptid, glukose, IGFBP-1, IGF-I, adiponectin, metabolitter, glukagon, Na+, K+, creatinin, carbamid, catecholaminer, hæmatokrit, hæmoglobin og albumin.

Eventuelle intravaskulære volumen forskelle mellem hver situation vil blive søgt elimineret gennem infusion af isotonisk NaCl.

Muskelbiopsi, fedtbiopsi samt metaboliske analyser udføres som beskrevet i det generelle metodeafsnit.

Herudover udføres undersøgelse af hjertets pumpefunktion:

*Ekkokardiografi:* Vingmed System FIVE systemet benyttes. De følgende parametre registreres: global uddrivningsfraktion (3D-ekko)^1^, regional wall motion score, (16 segment model), forkortningsfraktion, M-mode af venstre atrium, pulset Doppler i mitralannulus, regional longitudinel forkortningshastighed med vævs-Doppler (peak velocity, time to peak, strain og strain rate), diastolisk funktion (early diastolic myocardial velocity, E/A ratio, EdecT, IVRT, E/e’-ratio). Alle målinger beregnes som gennemsnit af 3-5 konsekutive hjerteslag. Vena cavas fylde og respiratoriske kolabering vil blive målt subxiphoidt som udtryk for hydreringstilstanden i hver situation.

*Arbejdstestning:* Arbejdstestning med cykelergometer under kontinuerlig EKG monitorering foretages på alle patienter. Belastningen øges 10W/minut. Hjertefrekvens, blodtryk, anaerob tærskel, maksimal iltoptagelse, non-invasivt cardiac output, maksimal arbejdskapacitet registreres.

*Non-invasivt Cardiac output:* INNOCOR apparaturet anvendes*.* Hvert tredje minut under arbejdstesten udføres genåndingstest hvor patienten i et lukket system indånder en blanding af atmosfærisk luft, ilt, lattergas og sulfur hexafluorid. Ved Fick´s princip kan lungernes gennemblødning og dermed Cardiac output beregnes.

Blod- og vævsprøver forventes undersøgt og analyseret umiddelbart efter prøvetagningen og destrueres herefter. Der oprettes derfor ikke en biobank.

## Statistik

Primært 3-vejs ANOVA, sekundært for diabetesgruppen parrede t-tests.

Der foreligger ikke data vedrørende intra- og interpersonel variabilitet for de primære signalvejs og proteom analyser, så der kan ikke foretages styrkeberegning.

Hvad angår den kardiologiske del kan der foretages følgende styrkeberegning:

n = 2 x *f*(*α,β*) x $\left(\frac{\sigma}{\delta}\right)^{2}$.

*α* er risikoen for type 1 fejl, *β* er risikoen for type 2 fejl, *σ* er standarddeviation og *δ* er den forskel vi ønsker at kunne detektere.

*Ekkokardiografi*: I reproducerbarheds-undersøgelser findes SD på global EF bestemt ved 3D-ekkokardiografi at være ca. 5 %. Hvis *α* er 5 % og *β* er 20 % er *f* 7.9. Hvis vi ønsker at kunne finde forskelle i EF på ca. 7 %, bliver n = 2 x 7.9 x (5/7)^2^ = 8.01.

*Arbejdskapacitet*: I undersøgelser findes SD på 10 % mht. exercise tid og maksimal iltoptagelse. Hvis vi ønsker at kunne finde forskelle i arbejdskapacitet på 14 %, bliver n = 2 x 7.9 x (10/14)^2^ = 8.01.

## Etik

Der henvises til afsnittet *Etiske Aspekter* under **Delforsøg 1**.

### Rekruttering og retningslinjer for skriftlig og mundtlig information

Forsøgsdeltagerne rekrutteres fra diabetes ambulatorierne på Regionshospitalet Silkeborg og Medicinsk Afdeling M, Århus Sygehus, NBG. De patienter, der opfylder in- og eksklusionskriterierne kontaktes pr. brev, indeholdende skriftligt informationsmateriale. I det skriftlige informationsmateriale vil patienten blive informeret om muligheden for at medbringe en bisidder til en mundtlig informationssamtale. Såfremt den potentielle forsøgsperson ønsker at deltage i forsøget eller blot ønsker at vide mere udfyldes en blanket, der sendes til investigator, hvorefter den mulige forsøgsperson kontaktes telefonisk af investigator og der vil her blive aftalt tid og sted for mundtlig information. De raske kontroller rekrutteres ved hjælp af opslag og kan rette henvendelse til investigator. Der vil da blive aftalt tid og sted for et informationsmøde, hvor der gives mundtlig information og skriftlig information udleveres. Den mundtlige information om undersøgelsen vil foregå i enerum og vil blive givet af professor, overlæge Niels Møller, overlæge Lotte Ørskov, læge Ulla Kampmann Opstrup eller læge Roni Nielsen. Der vil være mulighed for at stille spørgsmål og for at frasige sig viden om egen helbredstilstand. Folderen ”Dine rettigheder som forsøgsperson i et biomedicinsk forskningsprojekt” udgivet af Den Centrale Videnskabsetiske Komité, vil blive udleveret. Det vil blive understreget, at deltagelsen er frivillig og at tilsagn om deltagelse derfor kan trækkes tilbage når som helst, uden at dette på nogen måde vil påvirke læge-patient forholdet. Efter informationssamtalen vil der være betænkningstid inden indhentelse af skriftligt samtykke. Samtykkeerklæringen underskrives af forsøgspersonen og sendes til investigator.

Forsøgspersonerne har mulighed for at få yderligere oplysninger om projektet ved at ringe til den projektansvarlige.

Den mundtlige og skriftlige information gives i overensstemmelse med Forskningsstyrelsens Vejledning om information og samtykke ved inddragelse af forsøgspersoner i biomedicinske forskningsprojekter.

### Forudsigelige risici og ulemper

Blodprøvetagning er forbundet med lette smerter og der er en beskeden risiko for infektion og blodansamling ved indstiksstedet. Forsøgspersonerne vil blive orienteret om tegnene på infektion og instrueret i at kontakte en af de projektansvarlige læger og komme til vurdering på sygehuset. Der anvendes almindelig steril teknik. Det samlede blodtab andrager ca. 15 ml, hvilket ikke forventes at give symptomer og medføre utilpashed. I forbindelse med undersøgelserne på afdeling M’s laboratorium vil der blive lagt intravenøs adgang i 2 perifere vener, dette kan være forbundet med let smerte. Der er i meget sjældne tilfælde beskrevet infektion efter anlæggelse af sådanne katetre.

Muskel- og fedtbiopsier er forbundet med et vist ubehag, men foregår under lokalanalgesi. Ved biopsierne er der ligeledes en meget lille risiko for infektion eller blødning i vævet. Ved symptomer herpå skal forsøgspersonen ligeledes henvende sig hos den forsøgsansvarlige eller kontakte den vagthavende bagvagt på Medicinsk Afdeling M, Århus Sygehus. Man vil kunne opleve smerte fra biopsistedet i nogle dage efter biopsitagningen. For at minimere risikoen for komplikationer vil indgrebene blive foretaget af en erfaren læge.

Ultralydsscanning af hjertet foregår med gelé på huden og er smerte- og komplikationsfrit.

Arbejdstestning kan udløse brystsmerter hos patienter med Angina pectoris og kan i sjældne tilfælde udløse farlige rytmeforstyrrelser. Patienter i kendt højere risiko ved arbejdstest (kendt symptomatisk aortastenose) vil ikke blive inkluderet i studiet. Arbejdstestning er en rutinemæssig undersøgelse på alle Kardiologiske afdelinger og vurderes som sikker under forudsætning af hurtig adgang til avanceret genoplivningsudstyr. Dette udstyr er til stede ved testen. Alvorlige komplikationer ved arbejdstest angives til <1/10000^2^.

Under arbejdstesten udføres non-invasiv cardiac outputmåling ved genånding i pose. Denne procedure er ufarlig for patienten.

Kvinder i den fødedygtige alder skal være i behandling med p-piller, spiral eller anden sikker graviditetsforebyggende behandling i mindst 2 måneder før forsøget, under forsøget samt i 2 dage efter forsøgets afslutning.

Alle nævnte metoder er velkendte i forsøgslaboratoriet og af de projektansvarlige og vurderes at være absolut acceptable i relation til det overordnede formål.

### Fordele ved at deltage i undersøgelsen

Forsøgspersonen kan efter projektets afslutning få oplyst resultater af egne undersøgelser og svar på blodprøver, men der er ikke umiddelbart andre fordele ved at deltage i undersøgelsen. Forsøgspersonen vil imidlertid bidrage til ny vigtig viden, som han eller hun i sidste ende muligvis selv vil komme til at drage fordel af.

Kompensation

Deltagerne i undersøgelsen, der har sukkersyge, vil på individuel basis modtage godtgørelse for tabt arbejdsfortjeneste, samt refusion af udgifter til transport etc. Godtgørelsen for tabt arbejdsfortjeneste er skattepligtig.

De raske forsøgspersoner modtager et engangsbeløb på 1500 kr. for deltagelse i forsøget.

Beløbene og vil blive indsat på forsøgspersonens bankkonto efter undersøgelserne er afsluttet.

## Tidsforbrug

Det forventede tidsforbrug pr. undersøgelsesdag forventes at være ca. 7 timer. Der er 2 undersøgelsesdage. Herudover beregnes yderligere ½ undersøgelsesdag til de cardiologiske forundersøgelser. De 2 egentlige undersøgelsesdage udføres med 3-5 ugers interval.

Raske forsøgspersoner skal gennemgå én undersøgelsesdag af ca. 6 timers varighed.

## Arbejdsfordeling

Læge Ulla Kampmann Opstrup og professor, overlæge Dr.Med. Niels Møller vil stå for rekrutteringen af patienterne, mens læge Roni Nielsen vil assistere ved dette. Erfarne læger fra afdelingen vil være ansvarlige for biopteringen, mens læge Roni Ranghøj Nielsen står for de kardiologiske undersøgelser er læge Ulla Kampmann Opstrup ansvarlig for indsamling af de endokrinologiske data. Læge Ulla Kampmann Opstrup, læge Roni Ranghøj Nielsen, afdelingslæge Henrik Wiggers, afd. B, Skejby sygehus og professor dr.med. Hans Erik Bøtker,afd. B, Skejby sygehus og overlæge Dr. Med Niels Møller står for fortolkning af data. Analyser af ekkokardiografiske data vil blive søgt blindet.

## Opbevaring og behandling af data

Data beskyttes efter lov om behandling af personoplysninger og sundhedsloven. Data vil blive behandlet strengt fortroligt og vil kun blive publiceret i anonymiseret form. Data arkiveres på afdeling M, Århus Sygehus. Data skal arkiveres i 15 år efter undersøgelsens afslutning.

## Finansiering og Forsikring

Projektet finansieres af en puljebevilling fra Fødevareministeriet samt forbruger- og familieministeriet. Lønudgifter afholdes af Medicinsk Afdeling M.

Deltagerne er dækket i overensstemmelse med lov om patientforsikring samt lov om erstatning for lægemiddelskader.

Reference List

(1) Kim W, Sogaard P, Kristensen B, Egeblad H. Measurement of left ventricular volumes by 3-dimensional echocardiography with tissue harmonic imaging: A comparison with magnetic resonance imaging. *J Am Soc Echocardiogr* 2001; 14(3):169-179.

(2) Gibbons L, Blair SN, Kohl HW, Cooper K. The safety of maximal exercise testing

65. *Circulation* 1989; 80(4):846-852.
